# Management of Monogenic IL-1 Mediated Autoinflammatory Diseases in Childhood

**DOI:** 10.3389/fimmu.2021.516427

**Published:** 2021-03-18

**Authors:** Tatjana Welzel, Susanne M. Benseler, Jasmin B. Kuemmerle-Deschner

**Affiliations:** ^1^Autoinflammation Reference Center Tuebingen (arcT) and Division of Pediatric Rheumatology, Department of Pediatrics, University Hospital Tuebingen, Tuebingen, Germany; ^2^Pediatric Pharmacology and Pharmacometrics, University Children‘s Hospital Basel (UKBB), University Basel, Basel, Switzerland; ^3^Rheumatology, Department of Pediatrics, Alberta Children's Hospital (ACH), ACH Research Institute, University of Calgary, Calgary, AB, Canada

**Keywords:** treat-to-target, AID management, multidisciplinary team, disease activity, monitoring, autoinflammation

## Abstract

Monogenic Interleukin 1 (IL-1) mediated autoinflammatory diseases (AID) are rare, often severe illnesses of the innate immune system associated with constitutively increased secretion of pro-inflammatory cytokines. Clinical characteristics include recurrent fevers, inflammation of joints, skin, and serous membranes. CNS and eye inflammation can be seen. Characteristically, clinical symptoms are coupled with elevated inflammatory markers, such as C-reactive protein (CRP) and serum amyloid A (SAA). Typically, AID affect infants and children, but late-onset and atypical phenotypes are described. An in-depth understanding of autoinflammatory pathways and progress in molecular genetics has expanded the spectrum of AID. Increasing numbers of genetic variants with undetermined pathogenicity, somatic mosaicisms and phenotype variability make the diagnosis of AID challenging. AID should be diagnosed as early as possible to prevent organ damage. The diagnostic approach includes patient/family history, ethnicity, physical examination, specific functional testing and inflammatory markers (SAA, CRP) during, and in between flares. Genetic testing should be performed, when an AID is suspected. The selection of genetic tests is guided by clinical findings. Targeted and rapid treatment is crucial to reduce morbidity, mortality and psychosocial burden after an AID diagnosis. Management includes effective treat-to-target therapy and standardized, partnered monitoring of disease activity (e.g., AIDAI), organ damage (e.g., ADDI), patient/physician global assessment and health related quality of life. Optimal AID care in childhood mandates an interdisciplinary team approach. This review will summarize the current evidence of diagnosing and managing children with common monogenic IL-1 mediated AID.

## Introduction

Monogenic IL-1 mediated autoinflammatory diseases (AID) are rare, often severe disorders caused by variants in innate immunity genes resulting in a constitutive overproduction of pro-inflammatory cytokines (1, 2). Clinical characteristics can include recurrent fevers, inflammation of joints, eyes, skin, and serous membranes (3). Severe phenotypes can include inflammation of CNS, bones, inner ears with hearing loss, and kidneys (4, 5). Patients with AID frequently report fatigue, irritability, headache, abdominal pain, and musculoskeletal complaints (6–9). Characteristically, clinical symptoms are coupled with increased inflammatory markers, such as C-reactive protein (CRP) and serum amyloid A (SAA) (3). AA amyloidosis is a serious complication with a prevalence of up to 50% in untreated familiar Mediterranean fever (FMF) (10).

The genetic origin of IL-1 mediated AID was first determined for FMF in 1997 (11, 12). In 1999, mutations in the *TNFRSF1A* gene were shown to be associated with the Tumor necrosis factor (TNF) receptor-associated periodic syndrome (TRAPS) previously called Familial Hibernian fever (13–15). In the following disease-causing genes were identified for several AID including the Cryopyrin-Associated Periodic Syndromes (CAPS) and the Hyperimmunoglobulinemia D Syndrome (HIDS)/Mevalonate Kinase Deficiency (MKD). The AID spectrum is continuously expanding due to advancements in genomic technologies such as next generation-sequencing (NGS) (16, 17). Translational research has further advanced the pathophysiological understanding of AID.

The management of AID patients include early diagnosis, effective therapy, treat-to-target (T2T) strategies and standardized monitoring of disease activity and damage. Therefore, a multidisciplinary team approach and attention to disease-related psychosocial burden are important. This review will summarize the available evidence focusing on common monogenic IL-1 mediated AID including CAPS, HIDS/MKD, FMF, and TRAPS.

### Introducing the Inflammasome

**Figure 1:**
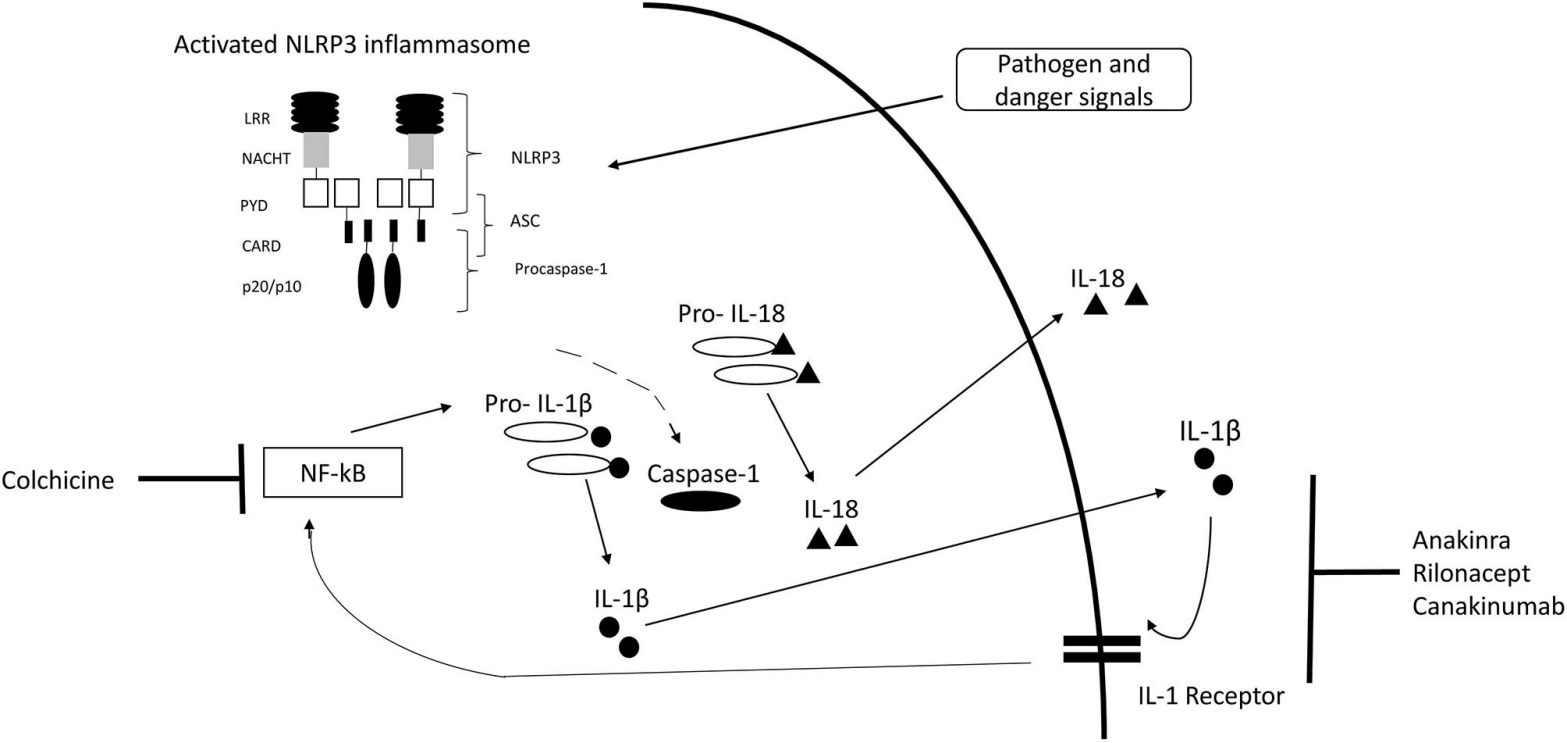
IL-1 inflammasome structure, cytokine release, and treatment target. Inflammasome formation is induced by a variety of triggers. Activated NLRP3 subsequently nucleates ASC forming filaments *via* PYD-PYD interactions and drives procaspase-1 filament formation through CARD-CARD interactions. Caspase-1 mediates cleavage of pro-IL-1β and pro-IL-18 to IL-1β and IL-18. [Modified from McCoy et al. (19), Lachmann et al. (20), Broderick (21)]. CARD, caspase activation and recruitment domain; LRR, leucine-rich-repeats; PYD, Pyrin domain; ASC, Apoptosis related speck-like protein containing CARD.

Inflammasomes are intracellular complexes controlling inflammation and immune cell activation triggered by a variety of exogenous and endogenous triggers (2, 18) (Figure 1). The nucleotide-binding domain-like receptor (NLR) family forms a group of proteins involved in the formation of inflammasome sensors (22). These contain a pyrin domain or a caspase activation and recruitment domain (22). One of the most prominent members of NLR families in monogenic AID is NLRP3 (22). Pyrin is another important inflammasome-forming protein (23). Inflammasome assembly (Figure 1) leads to the activation of caspase-1, which is able to process the inactive pre-cursor form of IL-1β to its mature bioactive form and induce its release (22, 24). IL-1β is one of the most prominent products of inflammasome activation and a key regulator of systemic inflammation (22). Genetic variants can alter proteins involved in the inflammasome pathways (25).

### Pathogenesis in Brief of Common IL-1 Mediated Aid

CAPS pathogenic gain-of-function variants in the *NLRP3* gene result in activation of the NLRP3 inflammasome with increased IL-1β secretion (26, 27). FMF is caused by autosomal recessive variants in the *MEFV* gene encoding for pyrin, a protein involved in the pyrin inflammasome (11, 12). HIDS/MKD results from loss-of-function mutations in the *MVK* gene, encoding for an enzyme of the isoprenoid biosynthesis (28, 29). The impaired isoprenoid biosynthesis leads to accumulation of mevalonate, shortage of end-products and reduced isoprenylated proteins (30, 31). Particularly, the shortage of geranylgeranyl-pyrophosphate affects small GTPases, resulting in IL-1β hypersecretion, activation of the pyrin inflammasome and the nuclear factor κB (NF-κB) pathway (32–34). TRAPS results from variants in the *TNFRSF1A* gene (14). The intracellular retention of the mutated receptor causes several pathological responses including autophagy, increased endoplasmatic reticulum stress, excessive mitochondrial reactive oxygen species and enhanced NF-κB activation with production of pro-inflammatory cytokines including IL-1 (35–37). Four distinctly different pathogenic variants in heterogeneous inflammatory pathways result in increased IL-1β release. It remains unclear, why the common feature of increased IL-1 β is associated with a heterogeneous clinical phenotype across diseases and even within each IL-1 mediated AID.

## Diagnosis

**Figure 2:**
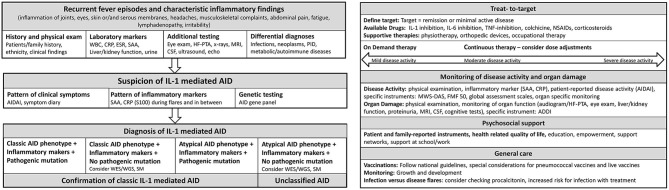
Flow sheet diagnostic steps and management of AID. AID, autoinflammatory disease; WBC, whole blood count; CRP, c reactive protein; ESR, erythrocyte sedimentation rate; SAA, Serum Amyloid A; HF-PTA, high frequency pure tone audiogram; MRI, magnet resonance imaging; CSF, cerebrospinal fluid; PID, primary immune deficiency; IL, Interleukin; AIDAI, Autoinflammatory Disease Activity Index; S100, S 100 proteins; WES, whole exome sequencing; WGS, whole genome sequencing; SM, somatic mosaicism; TNF, Tumor necrosis factor; NSAIDs, Nonsteroidal anti-inflammatory drugs; MWS-DAS, Muckle Wells Syndrome Disease activity score; FMF 50, Familial Mediterranean Fever 50 Score; ADDI, Autoinflammatory Disease Damage Index.

In 2012, Toplak et al. reported a medium diagnostic delay for AID of more than 7 years (range 0.3–76). There was a rapid increase in recognition after the first AID gene discovery in 1997 (38). Diagnosis of AID should be made as early as possible to prevent organ damage (39). In patients with suspected AID, a stepwise diagnostic approach should be performed, including patient and family history, ethnicity, physical examination, and inflammatory markers during febrile attacks and symptom free-intervals and genetic testing (40–42) (Figure 2). Other differential diagnosis such as immunodeficiencies, infections, autoimmune diseases and malignancies need to be excluded. Red flags are a family history of early hearing loss or renal transplants, Mediterranean background, fever periodicity and specific flare triggers, such as cold exposure.

### Clinical Aid Symptoms

Recurrent fever is a leading symptom varying in duration for different AID associated with specific clinical symptoms. The Autoinflammatory Disease Activity Index (AIDAI), a standardized symptom diary (43), captures AID characteristic symptoms and helps identify phenotypic patterns.

The spectrum of **CAPS** includes three phenotypes and their overlaps: familial cold autoinflammatory syndrome (FCAS), Muckle-Wells syndrome (MWS), and the neonatal-onset multisystem inflammatory disease (NOMID)/chronic infantile neurologic cutaneous and articular syndrome (CINCA) (44, 45). The FCAS phenotype is characterized by cold-induced neutrophilic dermatitis, fever, and chills (4). Patients with the moderate MWS may suffer from sensorineural hearing loss, urticaria-like rash and arthritis, while the severe NOMID phenotype is associated with aseptic meningitis, skeletal deformities and papilledema (4). MWS onset is often in early childhood, while NOMID is frequently recognized in the neonatal period (4).

Characteristic symptoms of **FMF** are recurrent fevers ranging 1–3 days, serositis, abdominal pain and/or pericarditis, arthritis, myalgia, and erysipelas-like erythema (46).

**HIDS/MKD** related features include malaise, fatigue, weight loss, lymphadenopathy, mucocutaneous involvement, musculoskeletal complaints, and gastrointestinal symptoms (47). Neurological involvement and life-threatening macrophage activation syndrome are observed.

**TRAPS** patients typically present with musculoskeletal complaints, abdominal pain, maculopapular and migratory rash, and periorbital edema (6). Serositis, pericarditis, arthritis and myalgia may be more prominent in adulthood, while abdominal pain is found more typically in childhood (6, 48). Furthermore, Gaggiano et al. highlight that high penetrance variants are associated with abdominal pain, amyloidosis and subclinical inflammation, whereas oral aphthosis is frequently observed in low-penetrance variants (48). TRAPS may start in adulthood, family history may then be non-contributory.

### Laboratory Investigations

CRP, SAA and complete blood count are considered first line laboratory parameters and should be tested during febrile attacks and in symptom-free intervals (42). Liver and muscle enzymes, renal function tests, and urine analysis including 24 h evaluation for proteinuria should be performed serially (Figure 2). Immunoglobulin D (IgD) and mevalonic acid levels may be useful in suspected HIDS/MKD (47).

### Functional Testing

AID can affect multiple organ systems and may result in organ damage. Functional testing is required at diagnosis and during monitoring (Figure 2). In suspected AID, a thorough physical examination including growth, development, musculoskeletal, neurological, and ophthalmologic examination should be performed (39). In suspected CAPS, high frequency audiograms (HF-PTA) including 0.5–10 kHz, formal cognitive testing, magnetic resonance imaging (MRI) brain, spinal tap with opening pressure, cerebrospinal fluid (CSF) cell count and protein, and a lesional skin biopsy should be considered (39). In patients with severe musculoskeletal involvement, x-rays and bone MRI are recommended (39). In HIDS/MKD, additional cognitive testing and muscle and liver enzyme monitoring are recommended (39). For FMF patients, particularly during colchicine treatment, renal and liver function tests should be monitored serially (49).

### Genetic Testing

Molecular testing should be performed, when the clinical phenotype, laboratory and functional tests are suggestive of AID (40). Sanger sequencing had been primarily utilized to identify AID-causing variants. In recent years, NGS-AID panels have become the gold standard (50). Complete coding sequences of AID genes are enriched in these panels. The Genetic Testing Registry database (https://www.ncbi.nlm.nih.gov/gtr/) provides an overview of available panels. The reported diagnostic yield of comprehensive gene testing panels seems to range between 21 and 32% (51, 52). Whole exome sequencing (WES) should be considered in patients with negative panel testing to improve the diagnostic yield preferably using a family-based trio approach (53). In addition, WES enables the discovery of novel variants in known AID genes and in those not yet associated with diseases. Whole genome sequencing (WGS) can identify deep intronic variants and mutations in non-coding regulatory regions and therefore increases the diagnostic yield (53). Additionally, it allows a much more reliable identification of copy number variations compared with WES (53).

Somatic mosaicism results from *de novo* post-zygotic mutations. AID-panel testing in these patients may be negative. AID onset and phenotype may be atypical. Saito et al. first identified somatic mutations causing NOMID (54). Subsequently, Tanaka et al. reported somatic mutations in 70% of previously mutation-negative NOMID patients (55).

The identified pathogenic variants may allow prediction of disease severity; for example FMF patients with homozygous M694V were found to be at high risk for a severe phenotype including early disease-onset (56). Similarly, in HIDS/MKD combined heterozygosity for p.V377I/p.I268T was shown to be a risk factor for AA amyloidosis (47). Genetic variants are typically described as “pathogenic,” “likely pathogenic,” “uncertain significance,” “likely benign,” and “benign” (57). Some platforms can assist clinicians and geneticists in determining the pathogenicity of variants including the MOLGENIS platform (58). The Infevers database is an exhaustive registry of sequence variants identified in AID related genes (59).

### Diagnostic and Classification Criteria

Diagnostic criteria are used to guide the care of individual patients, establish the correct diagnosis, and start targeted treatment (60). The diagnostic criteria for CAPS mandate raised inflammatory markers (CRP/SAA) *plus* at least two of the following symptoms: neutrophilic dermatitis, cold-triggered episodes, sensorineural hearing loss, musculoskeletal symptoms, chronic aseptic meningitis, and skeletal abnormalities (61). These criteria enable physicians to make a CAPS diagnosis without mandating evidence of a disease causing variant (e.g., in case of low-penetrance variants). For FMF, the first Tel Hashomer criteria were proposed for adult patients in 1967 (62). In 1997, Livneh et al. proposed a set of diagnostic FMF criteria based on the presence of one major or two minor criteria, or one minor plus five supportive criteria (63). The major criteria are typical attacks (lasting 12–72 h, >3 attacks, fever > 38°C) with any one of peritonitis, pleuritis/pericarditis, monoarthritis or unilateral “orchitis”, erysipelas-like eruption in the calf, and/or symmetric myalgia with extreme tenderness in the lower extremities. The minor criteria were defined as incomplete attacks, exertional leg pain and favorable response to colchicine. In 2009, Yalçinkaya, Ozen et al. proposed the pediatric FMF criteria requiring ≥2 of the following characteristics: fever, abdominal pain, one-sided chest pain and arthritis, each lasting 6–72 h, ≥3 attacks, and family history of FMF (64), allowing to make a clinical FMF diagnosis in case of inconclusive/negative genetics and are useful in selection which patients should be genetically tested. These criteria were developed in a Turkish population. Subsequently, Demirkaya et al. compared the performance of these existing criteria in the pediatric AID cohort of the Eurofever registry (65). The Yalchinkaya-Ozen criteria yielded a higher sensitivity (87.4%) than 1967 and 1997 criteria. The authors suggest that they can be used for FMF diagnosis in pediatric patients from European and eastern Mediterranean region (65). Classification criteria are primarily used to define patients cohorts for research (60). In 2019, Gattorno et al. published validated evidence-based classification criteria for hereditary AID including CAPS, HIDS/MKD, FMF, and TRAPS with pathogenic/likely pathogenic variants, low-penetrance variants and without genetic testing/findings (66).

### Diagnostic Uncertainty

While advanced genetic testing may establish a diagnosis in some patients, testing may still be negative, inconclusive or even misleading (1, 52, 67, 68). Therefore, the correlation of clinical phenotype and genetic result is critical (67). Low-penetrance variants in AID genes can be present in the general population. As some of these low-penetrance variant carriers nevertheless express AID symptoms unlike the known classical phenotype in confirmed pathogenic variants (69), it might be possible that these are mediated by different pathways parallel to the caspase-1 activation (70). Moreover, low-penetrance variants may confer an increased susceptibility to inflammation (71).

## Effective Aid Therapy: Treat-to-Target (T2T)

Therapy is comprehensive including medication, psychosocial support, physiotherapy and supportive care such as hearing aids (Figure 2). Traditional symptomatic therapy consisted of non-steroidal anti-inflammatory drugs (NSAIDS) and glucocorticoids, which often shortened disease flares but can increase their frequency (39, 49). Today, IL-1 inhibitors play a pivotal role and evidence-based AID treatment plans are available (39, 49).

Targeting of inflammatory pathways enables T2T strategies (19, 72) (Figure 1). Key component of T2T is the definition of a target such as remission or minimal disease activity. Standardized serial assessments are required to determine, if the target is achieved (72) (Figure 2). Different levels of disease activity require different treatment approaches (39, 72, 73). Frequently, dose adjustments are required, particularly in children and in severe disease subtypes (74, 75).

### Colchicine

Colchicine is the established first-line FMF therapy with favorable response and risk reduction of AA amyloidosis (49, 76). Colchicine should be started as early as possible (49). Colchicine is metabolized by CYP3A4 enzymes and the P-glycoprotein (P-gp) efflux transporter, therefore, concomitant treatment with CYP3A4 and/or P-pg inhibitors should be avoided (77, 78).

### IL-1 Targeting Drugs

IL-1 inhibition has been shown to be safe and effective in controlling inflammation in CAPS, TRAPS, and MKD/HIDS (75, 79–82). It is a valid therapeutic strategy in FMF patients with colchicine-intolerance/resistance (83–86). Currently, three IL-1 inhibitors are approved by the US Food and Drug Administration for AID.

Long-term efficacy and safety of the short-acting recombinant IL-1 receptor antagonist **anakinra** has been confirmed in several studies (79, 86–90). Anakinra is administered daily subcutaneously and blocks the binding of IL-1α and IL-1β to the IL-1 receptor. In a study of 43 CAPS patients treated with anakinra up to 5 years, serious adverse events reported most frequently were pneumonia and gastroenteritis (79). There is evidence that pediatric patients with undifferentiated AID may also benefit from anakinra (91).

The recombinant soluble IL-1 receptor **rilonacept** binds to IL-1α and IL-1β. Weekly subcutaneous administration has shown a good safety and efficacy profile (92).

**Canakinumab** is a fully humanized anti-IL-1β monoclonal antibody selectively binding soluble IL-1β. It has to be administered every 4–8 weeks subcutaneously. Several studies confirmed long-term efficacy and safety (93–98). Brogan et al., reported complete response to canakinumab in 17 CAPS patients <5 years of age (71% MWS, 24% NOMID, 6% FCAS) (96). Open label observations suggest that children require higher doses up to 8 mg/kg/4 weekly to achieve remission, particularly in severe CAPS phenotype (94). Recently, efficacy and safety of canakinumab was demonstrated for FMF, TRAPS and HIDS/MKD (75, 99). In patients with HIDS/MKD dose adjustment is frequently needed (75). The rate of serious infections was 7.4/100 patients-years in 181 patients with TRAPS, HIDS and CAPS (75).

### Alternatives

In a prospective open-label dose escalation study, etanercept reduce symptoms and inflammatory markers in a dose-dependent manner in TRAPS (100). FMF patients with chronic arthritis and sacroiliitis can benefit from TNF-inhibition (101). IL-6 inhibition may be promising in TRAPS patients (102) and HIDS/MKD, particularly when refractory to anakinra/etanercept (103–105). Hematopoietic stem cell transplantation has been performed in refractory HIDS/MKD patients (39).

### Psychosocial Needs in Aid

The care of patients with AID should include psycho-social support, as AID affect all areas of life (106) (Figure 2). AID are associated with depression, lower health related quality of life, anxiety and social isolation (107–110). Patients/parents have to deal with work/school-related challenges because of frequent sick-leaves (107). Long-term management should take psychological factors such as illness beliefs, coping strategies and the distribution of dependency into account (108). Patient support networks can provide important support (111).

## Monitoring of Aid Activity and Damage

Regular monitoring of disease activity is crucial (39). This includes physical examination, measurement of height and weight, neurological and musculoskeletal examination, and determination of SAA and CRP levels to detect ongoing inflammation (Figure 2). Repeatedly increased SAA levels between AID flares may indicate a significant risk for AA amyloidosis. Monitoring of SAA and S100 proteins may detect subclinical diseases activity, particularly in FMF (112, 113).

The validated patient-reported AIDAI is a simple tool for assessing disease activity (43). The AIDAI contains 13 items addressing fever (>38°C), overall symptoms, specific AID symptoms, and use of NSAIDs (43). The clinical symptoms are dichotomous and scored as 0 (absent) or 1 (present) (43). The maximum score per day is 12 with a cumulative monthly score ranging from 0 to 372 (43). The clinical meaningful threshold indicating active AID is a score of at least 9 (43). The Autoinflammatory Disease Damage Index (ADDI) is a reliable instrument to assess disease-related organ damage in FMF, CAPS, TRAPS and HIDS/MKD (114). ADDI consists of 18 items grouped in eight categories of reproductive, renal/amyloidosis, developmental, serosal, neurological, auditory, ocular, and musculoskeletal damage (114, 115). Damage is defined as persistent or irreversible change in structure or function present for at least 6 months (114, 115). The ADDI can be used to monitor structural damage in individual patients, and allows outcome analysis and comparison of damage accrual in clinical trials (114).

## Infections and Vaccines

Patients can experience febrile inflammatory episodes not primarily related to their AID. Particularly in atypical AID-flares, infections have to be excluded (39). While CRP does not discriminate between infection and flare, procalcitonin (PCT) may be a promising marker (116). Some infectious diseases are preventable by vaccination. However, both vaccination and infection may trigger flares, particularly in HIDS/MKD (117). Patients with CAPS may develop severe local and systemic inflammatory reactions after pneumococcal vaccination (118, 119). The 13-valent pneumococcal conjugate vaccine appears to be more favorable compared to the polysaccharide vaccine (119). In general, vaccination recommendations for patients with immunosuppressive therapy and inflammatory rheumatic diseases can be used for AID, where inactive vaccines are considered as safe and are recommended to national vaccination guidelines (120–123) (Figure 2). In accordance with the recommendations of the European League Against Rheumatism, annually influenza vaccinations are recommended for AID patients and immunosuppressive therapy (120, 124). Live -vaccines should be avoided/has to be considered individual for the patient (121, 122, 124).

## Summary

AID are rare diseases associated with the risk of severe morbidity, mortality and reduced health-related quality of life. The increasing number of somatic mosaicisms and low-penetrance variants make the diagnosis of these potential live-threatening diseases challenging. A standardized diagnostic approach for suspected AID should include the clinical phenotype, inflammatory markers, functional, and genetic testing (Figure 2). AID panels should be performed and may need to be supplemented with WES/WGS. The management of AID mandates a multidisciplinary team and psychosocial support. Medication should be tailored individually using T2T strategies. In IL-1 mediated AID, colchicine, and IL-1 inhibition are effective. Alternative therapies including IL-6 inhibition and TNF-blockade can be beneficial. Regular target evaluation and standardized monitoring of disease activity and organ damage is important. Vaccines should be administered according to national vaccination guidelines, respecting general vaccination recommendations for patients with rheumatic diseases.

## Author Contributions

TW, SB, and JK-D conceived the concept of the manuscript, wrote the manuscript, drafted the work, and reviewed the article critically. All authors have provided approval for publication.

## Conflict of Interest

SB participated on Add-boards from Sobi and Novartis. JK-D received speaker honoraria and grant support from Sobi and Novartis. The remaining author declares that the research was conducted in the absence of any commercial or financial relationships that could be construed as a potential conflict of interest.
